# Pharmacological, Technological, and Digital Innovative Aspects in Rhinology

**DOI:** 10.3389/falgy.2021.732909

**Published:** 2021-12-15

**Authors:** Rosanna Ruggiero, Giovanni Motta, Giuseppe Massaro, Concetta Rafaniello, Alberto Della Corte, Antonella De Angelis, Annalisa Capuano, Gaetano Motta, Francesco Rossi

**Affiliations:** ^1^Campania Regional Centre for Pharmacovigilance and Pharmacoepidemiology, Naples, Italy; ^2^Department of Experimental Medicine, Section of Pharmacology “L. Donatelli”, University of Campania “Luigi Vanvitelli”, Naples, Italy; ^3^Department of Mental Health and Public Medicine, Section of Otorhinolaryngology—Head and Neck Surgery, University of Campania “Luigi Vanvitelli”, Naples, Italy

**Keywords:** pharmacological innovation, digital innovation, technological innovation, monoclonal antibodies, dupilumab, mepolizumab, chronic rhinosinusitis (CRS), rhinology

## Abstract

Innovation refers to the introduction of a product, a process, a service or a solution resulting in something new or significantly improved compared to the already available alternatives. In the clinical context, it is strictly related to the identification of a new added value in terms of quality, therapeutic efficacy and safety. Over the years several innovative approaches have been introduced in the clinical practice, revolutionizing the treatment and the management of important rhinologic conditions. Innovative tools, including new drugs, biomaterials, and mobile applications seem to be able to improve the clinical outcomes and the quality of life of many patients affected by (often relapsing) rhinologic diseases. Among the main modern pharmacological innovations, mention must be made of the biological drugs like monoclonal antibodies (mAbs). Recently, new mAbs have been introduced and investigated as useful arms in the treatment of some inflammatory/infectious or oncological diseases affecting the nasal cavities and paranasal sinuses. The already approved or still investigated mAbs work inhibiting different type 2 inflammation pathways, including those mediated by IgE (omalizumab), IL-4/IL-13 (dupilumab), and IL-5 (mepolizumab). Moreover, considering the higher expression of PD-L1 in nasopharyngeal carcinoma, the use of PD-1 inhibitors, such as nivolumab, or a dual CTLA-4/PD-1 blockade (ipilimumab plus nivolumab) appear to be an effective strategy for the treatment of this cancer form. The implants with bio-absorbable biomaterials represent new interesting available technological innovations. Moreover, advanced technologies such as the artificial intelligence, the machine learning as well as the augmented or virtual reality have also proved useful in rhinologic field with main impacts on precision medicine and surgery. Finally, the development and use of mobile-Health tools represent a winning strategy in monitoring of the therapy success, safety and tolerability as well as the progress of chronic disease including chronic rhinosinusitis with nasal polyps. Supporting the research of innovative tools and strategies (including pharmacological, technologic, or digital ones) is essential to improve the management of chronic diseases that significantly affect the patients' quality of life.

## Introduction

Innovation refers to the introduction of a product, a process, a service or a solution resulting in something new or significantly improved compared to the already available alternatives (1). Regarding drugs, innovation is strictly related to the identification of an added value in terms of quality, therapeutic efficacy and safety, established on the basis of the results emerging from randomized clinical trials. The research and development of innovative medicines is fundamental to address persisting unmet therapeutic needs. Both the Food and Drug Administration (FDA) and the European Medicines Agency (EMA) support it through the so-called Early Access Programs (EAPs) (2). In Italy, the marketing authorization of a medicinal product, the possible recognition of its reimbursement or its innovativeness are not automatically consequential. Even if substantially based on the same evidence, they represent three distinct procedures. Using a multidimensional approach, the therapeutic need, the added therapeutic value and the quality of evidence represent the three variables to consider in the innovative evaluation (3).

Current innovations can involve three overlapping domains: *pharmacological*, referred to the discovery of new molecules with innovative mechanisms of action or the recognition of new therapeutic indications for already authorized drugs, *technological* considering new release/administration systems of already available drugs, and *digital* such as new medical software or applications. Today, we are experiencing a renaissance of innovation. Recently, several innovative drugs have been approved and introduced into clinical practice, revolutionizing the treatment of important diseases, such as hepatitis C or several types of cancer. Still other new innovative drugs are going to be authorized (new monoclonal antibodies for the treatment of Alzheimer's, neoplasms, asthma, chronic obstructive pulmonary disease and cardiovascular diseases). Pharmaceutical innovation allowed important therapeutic results. Thanks to innovative drugs, it has been possible to increase the life expectancy of many patients, transforming lethal pathologies into chronic ones. The increase in the 5-year mortality rate for various oncological diseases as well as the reduction in the mortality rate of HIV/AIDS are unequivocal examples. Likewise, innovative drugs are enabling continued advances in the management of the COVID-19 pandemic. Such progress is also made possible by the introduction of innovative research models. Over the years, the Research and Development sectors of biopharmaceutical companies evolved from a *closed innovation* model, where innovation was centralized within the company, to arrive at an *open innovation* enabling collaborations outside the company. Today, the companies are increasingly concentrated in *network innovation* activities, i.e., the acquisition of research and development services (R&D extra muros), machinery and software aimed at innovation and skills from other companies or institutions (4).

**Figure 1:**
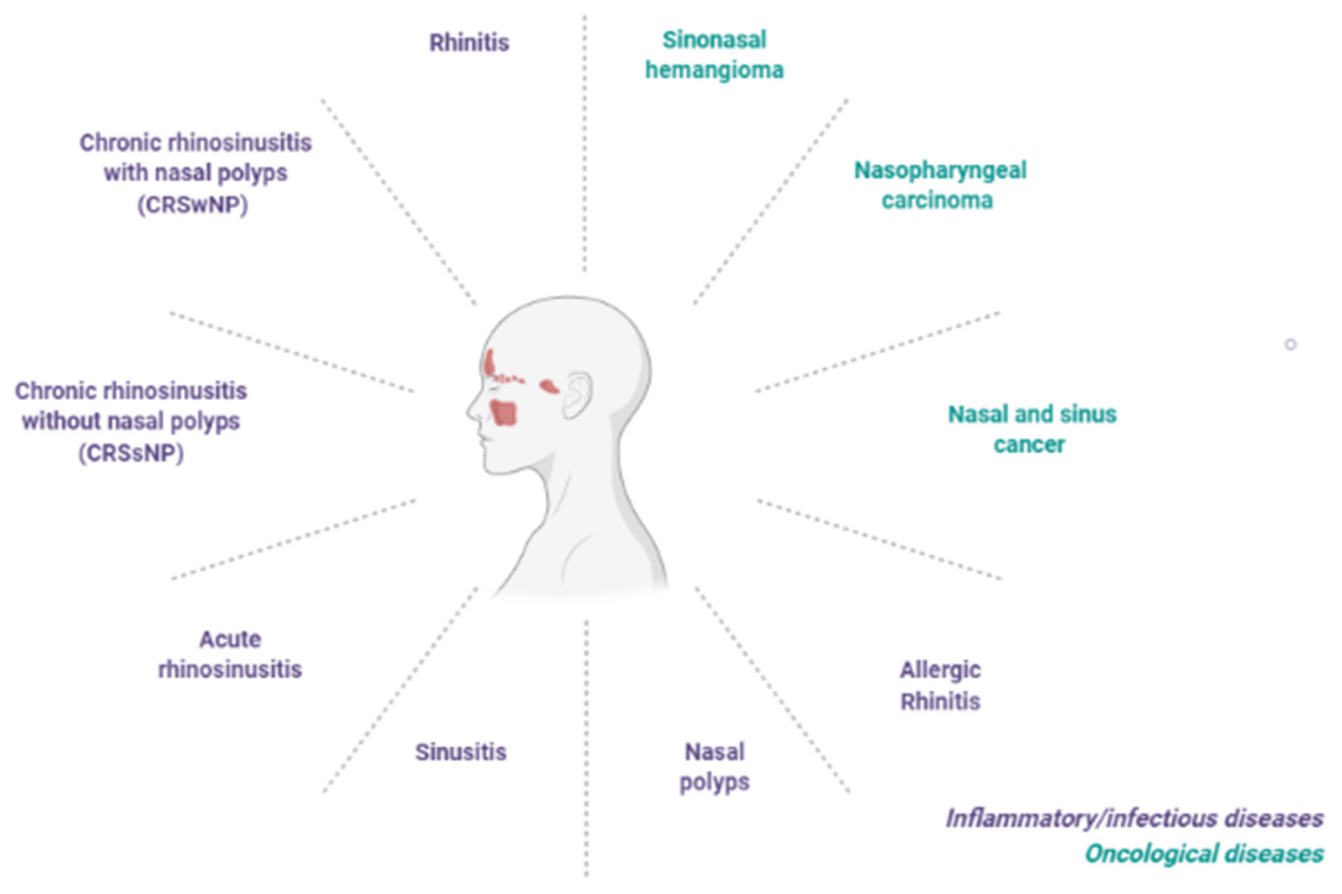
Main inflammatory/infective and oncological conditions affecting the nose and sinus cavities or upper respiratory tract.

Pharmacological innovation has also involved the field of rhinology. A variety of conditions affect the nose and sinuses, including inflammatory diseases, i.e., rhinitis, sinusitis, nasal polyposis, up to tumors of the nasal cavities and paranasal sinuses (Figure 1). The traditional treatments for inflammatory rhinological diseases include symptomatic therapies, based on antihistamine drugs and nasal decongestants, and disease modifying treatments, such as topical corticosteroids. New innovative drugs are able to improve clinical outcomes and quality of life of many patients affected by (often relapsing) rhinological diseases. Some of the main advances achieved in terms of pharmacological, technological as well as digital innovation applied to the field of rhinologic diseases are described below.

## Pharmacological Innovation

Among the main modern pharmacological innovations, biological drugs are revolutionizing the treatment of several pathologies, finding application in many therapeutic fields, including the rhinologic one (5). A biological drug is characterized by an active substance (generally a high molecular weight protein) produced by a living organism (microorganisms or animal cells) or using a biological source through the use of recombinant DNA techniques (biotechnological drugs). Biologics are more complex molecules than chemical drugs. Their major complexity is associated with an increase in their structural dimensions. Among the main classes or categories of biologicals, monoclonal antibodies (mAb) are worthy of note (6). Over the years, the approved mAb therapies have seen incredible growth, evidenced by the fact that in 2018, globally, six out of 10 best-selling drugs were mAbs (7). Today, in the COVID-19 pandemic context, mAbs represent an important part of the therapeutic armamentarium useful against SARS-CoV-2. Since they are able to block the viral attachment of SARS-CoV-2 to host cells, they seem to be promising tools in patients at early stage of COVID-19, preventing its progression and reducing the morbidity and mortality of infection such as the frequency of hospitalizations (8, 9). Overall, excellent efficacy profiles and lower frequency of adverse reactions characterize these drugs. Recently, some mAbs have been introduced and investigated as useful arms in the treatment of some inflammatory/infectious or oncological diseases affecting the nasal cavities and paranasal sinuses.

### Innovative Drugs for the Treatment of Inflammatory/Infectious Rhinological Conditions

**Figure 2:**
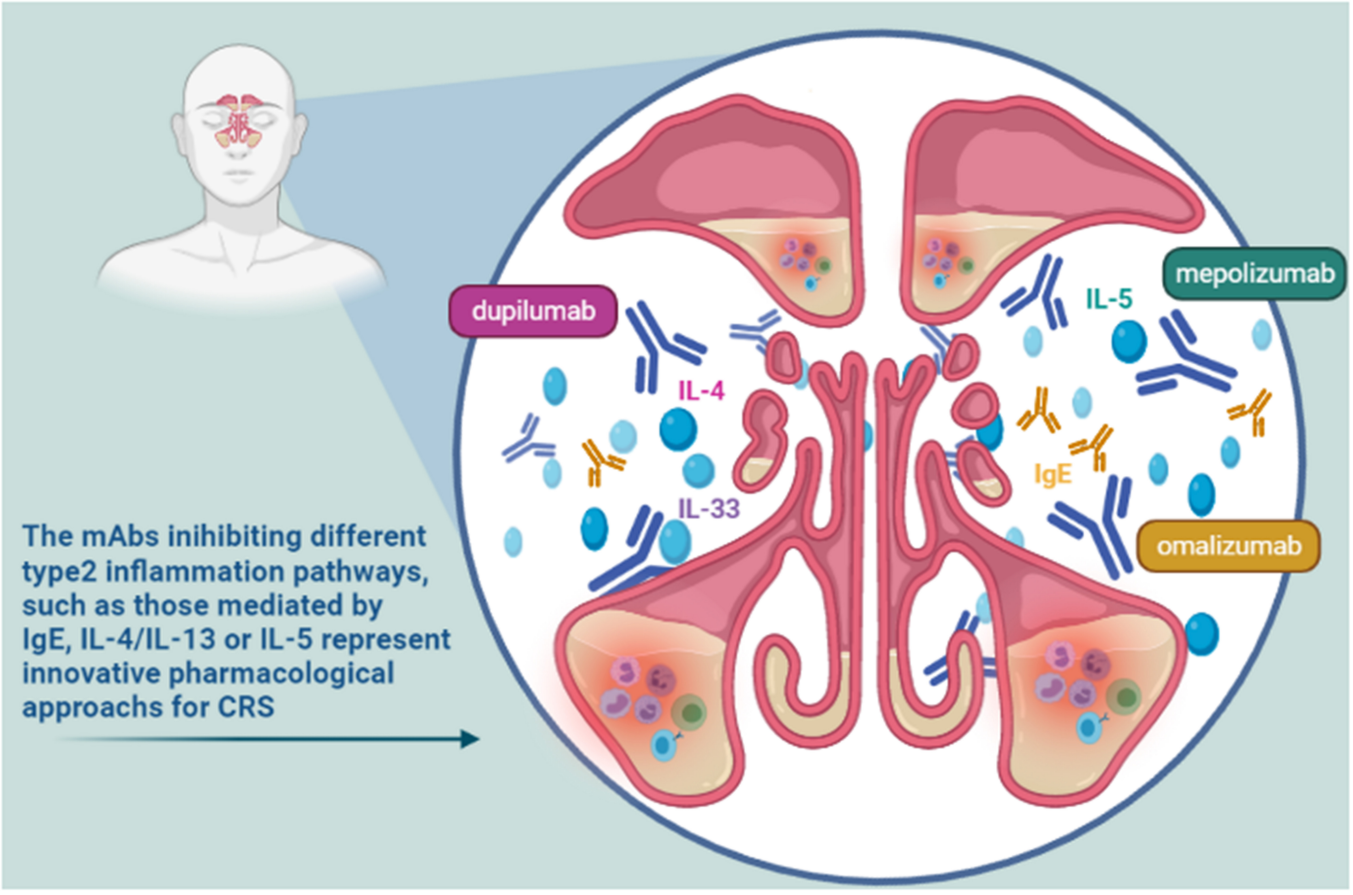
Some target pathways target of mAbs effective in chronic rhinosinusitis (CRS).

The rhinological diseases sharing inflammatory features, such as airway eosinophilia, local IgE formation, and a TH_2_ cytokine profiles, are evaluated as possible indications for some mAbs (10). Nasal polyps (NP), asthma, rhinitis and sinusitis, individually and in their various possible associations, represent some of these clinical challenges. Moreover, these pathologic conditions are often comorbid, with serious effect on the quality of life of patients. The already approved or still investigated mAbs work inhibiting different type 2 inflammation pathways, including those mediated by IgE, IL-4, IL-5, and IL-13 (Figure 2). Such mAbs represent useful tools for a precision medicine approach in the evaluation and management of severe chronic inflammatory conditions of upper respiratory tract (11), such as chronic rhinosinusitis (CRS) (11, 12). CRS is characterized by local inflammation of the upper airways and sinuses which persists for at least 12 weeks (13). It affects ~3% of the population worldwide (14). It is often associated with several co-morbidities including nasal polyps, asthma, acute infection, and obstructive sleep apnea. Based on the associated presence of nasal polyps, CRS was classified into two phenotypes: CRS with nasal polyps (CRSwNP) and CRS without nasal polyps (CRSsNP) (15). The investigated mAbs seem to be particularly effective in the management of CRSwNP. This phenotype is predominantly an adult disease, with an average onset between 40 and 60 years old, frequently associated with severe asthma. It is difficult to treat, as often relapsing, even after surgery. CRSwNP is a debilitating disease accompanied by complete anosmia, headaches, often requiring chronic therapies with douching, topical corticosteroids, systemic corticosteroids and antibiotics, plus repeated surgical polypectomies to control the disease (16). This disease shows a substantial clinical and economic burden, significantly impacting on patients' lives and often causing missed work, and hospitalizations (17). Most patients affected by CRSwNP show a type 2 inflammatory form in the nasal and paranasal sinus mucosa. In particular, the degree of type 2 inflammation is correlated with disease severity of CRSwNP. On the other hand, in about 80% of patients chronic rhinosinusitis is characterized by the absence of nasal polyps. This disease phenotype is primarily associated to type 1 inflammation (13). Neverthless, increased levels of IL-4, IL-5, and IgE have been recently observed also in some patients with CRSsNP. So, some mAbs targeting on these pathways might be effective also in this patients population (18). In the light of this recent evidence, in the European Position Paper on Rhinosinusitis and Nasal Polyps (EPOS) 2020 the previous phenotype-based classification of CRS has been replaced, highlighting the anatomic distribution (localized or diffuse) and endotype dominance (type 2 or non-type 2) (19). To date, IgE, IL-4, and IL-5 represent the main targets of identified effective mAbs. Moreover, other possible targets for biological treatment of eosinophil and mast cell-related diseases such as CRSwNP seem to be IL-33, IL-17, thymic stromal lymphopoietin (TSLP) (Table 1). Overall, biologic therapy with mAbs targeting IgE (omalizumab), IL-4Rα (dupilumab), or IL-5 (reslizumab, mepolizumab) led to the improvement of several clinical outcomes, including reduction size of nasal polyps, favorable impact on quality of life, nasal airflow capacity and smell. Overall, the use of these agents was found to be safe and well-tolerated (20). Recently, a Cochrane Review focusing on the clinical management of patients with NP and CRS evaluated studies referred to three main biologics dupilumab, mepolizumab, and omalizumab. Disease-specific health-related quality of life (HRQL), disease severity and serious adverse events were the primary outcomes. All the patients enrolled in the included studies were using topical nasal steroids. According to the results (summarized in Table 2), dupilumab represents the mAb inducing more improvement in all considered primary outcomes (16).

**Table 1:** Main pathways target of recent innovative pharmacological treatments, their approved indications in Europe and United States and study phase for other inflammatory/infective sinonasal conditions.

**Pathways**	**mAbs**	**Target**	**Approved indications in EU by ema**	**Approved indications in us by FDA**	**studies phase in NP, CRS, or CRSwNP, CRSsNP, AR**
IgE	Omalizumab	IgE	- CRSwNP - CIU - Allergic asthma	- Nasal polyps - CIU - Allergic asthma	Phase 3 (AR)
	Ligelizumab	IgE	-	-	No trials yet
	Quilizumab	IgE	-	-	Phase 1 (AR)
IL-5	Mepolizumab	IL-5	- Eosinophilic asthma	- Eosinophilic asthma - HES - EGPA	Phase 3 (NP)
	Reslizumab	IL-5	- Eosinophilic asthma	- Eosinophilic asthma	Phase 3 (CRS)
	Benralizumab	IL-5Rα	- Eosinophilic asthma	- Eosinophilic asthma	Phase 3 (NP) Phase 3 (CRSwNP)
IL-4/IL-13	Dupilumab	IL-4Rα	- Atopic dermatitis - Asthma - CRSwNP	- Atopic dermatitis - Asthma - CRSwNP	Phase 3 (CRSnNP) Phase 4 (CRSwNP)
	Tralokinumab	IL-13	- Atopic dermatitis	- Atopic dermatitis	No trials yet
	Lebrikizumab	IL-13	-	-	No trials yet
IL-17	Brodalumab	IL-17RA	- Psoriasis	- Psoriasis	No trials yet
IL-33	Etokimab	IL-33	-	-	Phase 2 (CRSwNP)
TSLP	Tezepelumab	TSLP	-	-	Phase 3 (CRSwNP)

*CIU, chronic idiopathic urticarial; AR, Allergic Rhinitis; NP, nasal polyps; TSLP, thymic stromal lymphopoietin; EGPA, eosinophilic granulomatosis with polyangiitis; HES, hypereosinophilic syndrome; CRS, chronic rhinosinusitis; CRSsNP, chronic rhinosinusitis without nasal polyps; CRSwNP, chronic rhinosinusitis with nasal polyps, EU, Europe; US, United States; EMA, European Medicines Agency; FDA, Food and Drugs Administration*.

**Table 2:** Main results of a recent Cochrane Review on the clinical management of patients with NP and CR with biologics.

**mAb compared to placebo**	**Disease-specific HRQL**	**Disease severity**	**Serious adverse events**
Dupilumab (anti IL-4)	Improve	Results in a reduction	May result in a reduction in number
Mepolizumab (anti IL-5)	May improve	Very uncertain difference	Very uncertain difference
Omalizumab (antiIgE)	Probably improve	No evidence	Very uncertain difference

#### mAbs Targeting the IgE Pathway

***Omalizumab*** is an anti-IgE humanized mAb produced by recombinant DNA technology. It was already approved in 2002 for the treatment of allergic asthma in adults, adolescents and children aged ≥6 years. Since February 2014, it was also approved for the treatment of chronic spontaneous urticaria in patients aged ≥12 years. Finally, in July 2020, omalizumab obtained another extension of indication as add-on therapy for the treatment of adults (age ≥18 years) with severe CRSwNP for whom only intranasal corticosteroid therapy does not provide adequate disease control (21). Omalizumab dosing reflects the personalized approach to which biological therapy is aimed. In fact, as reported in the Summary of Product Characteristics, it is determined considering the baseline serum IgE level (UI/mL) and body weight (Kg) of each patient. Based on these determinations, the dosing (from 75 to 600 mg) and the time intervals for its subcutaneous administration (every 2 or 4 weeks) are identified^1^. Binding selectively to IgE, omalizumab reduces the concentration of free IgE in blood and in tissue, surface IgE on basophils and mast cells and, consequently, blocks the effects of IgE on dendritic cells^1^. IgE is involved in some biological functions and mechanisms relevant for several diseases, including allergic rhinitis and nasal polyposis. Overall, omalizumab treatment is able to induce a reduction of nasal polyp size, an improvement of symptoms and the inhibition of underlying type 2 inflammation^2^. Several randomized, double-blind, placebo-controlled trials such as real-life studies showed omalizumab efficacy in the disturbances of nasal and/or sinusal mucosa. In particular, the results of a randomized, double-blind, placebo-controlled study showed omalizumab efficacy in improving airway symptoms (including nasal congestion, anterior rhinorrhea, loss of sense of smell, wheezing, and dyspnea) and quality-of-life scores in patients with nasal polyps and comorbid asthma. Its clinical efficacy occurred irrespective of the presence of allergy (10). Moreover, according results of a recent real-life study, omalizumab was able to treat both CRSwNP and asthma. The induced improvements in CRSwNP control were rapid and similar to that obtained with upper airway surgery (17). Finally, a recent study aimed to analyse in a real-life setting the therapeutic outcomes of mAb treatments, including omalizumab, was published. Moreover, the authors tried to identify possible predictive biomarkers for successful therapy. Their results confirmed the biologicals as promising treatment option of CRSwNP, especially in severe cases not responding to conventional therapy (22). Ligelizumab and quilizumab are other anti-IgE mAbs, mainly investigated as treatments for chronic spontaneous urticaria. Actually no trials for ligelizumab in rhinologic diseases have been yet initiated, while the evaluation of quilizumab efficacy in patients with allergic rhinitis is still in its early stages (Table 1) (23).

#### mAbs Targeting the IL-5 Pathway

IL-5 is another key driver of local type2-inflammation, produced by Th2 cells and group 2 innate lymphoid cells (ILC2s), stimulating the production, activation and maturation of eosinophils (24, 25). Approximately 85% of nasal polyps (NPs) are characterized by prominent eosinophilia. So, IL-5 inhibition with specific mAbs represent an innovative therapeutic approach in patients with NP or CRSwNP (26). To date, three mAbs targeting IL-5 (*mepolizumab* and *reslizumab*) or α-subunit of its receptor (*benralizumab)* have been developed for clinical use. Considering the important role of IL-5 in the development of bronchial hyper-responsiveness, all three mAbs have been evaluated in large-scale clinical trials as treatment for severe asthma. However, major studies evaluating the efficacy in patients with NP and CRSwNP have been conducted for mepolizumab. According the results of a meta-analysis, anti-IL5 therapy with mepolizumab induces a reduction in nasal polyp score in patients with CRSwNP (27).

**Mepolizumab** is a humanized mAb that binds IL-5, preventing its interaction with the α-chain of the IL-5 receptor (IL-5Rα). It was authorized by the EMA and the FDA in 2015 as an add-on treatment for asthma (28). Mepolizumab is innovative also for its pharmaceutical form of pre-filled syringe or pre-filled pen authorized by the EMA in 2019 representing the first European biologic drug for which self-administration in severe eosinophilic asthma was possible (29). In 2020, the regulatory approval for new additional indications for mepolizumab was submitted to EMA. These included three other eosinophil-driven diseases such as CRSwNP, hypereosinophilic syndrome (HES), and eosinophilic granulomatosis with polyangiitis (EGPA) (29). In the United States, mepolizumab has been already approved as treatment for adult patients with EGPA and represents the first and only biologic treatment for HES approved by the FDA. Moreover, it still waiting for FDA authorization as treatment for CRSwNP. Mepolizumab efficacy in nasal or sinus disturbances has been investigated in several studies. Already in a first randomized, double-blind, placebo-controlled study emerged that mepolizumab reduced the need for surgery at Week 25 and induced a greater improvement in symptoms compared to placebo. For this study patients were enrolled with recurrent eosinophilic nasal polyposis receiving topical corticosteroids and who required surgery. Mepolizumab's efficacy was accompanied with a safety profile comparable with placebo (30). Moreover, the efficacy and safety of mepolizumab as treatment of recurrent, refractory severe bilateral CRSwNP in adult patients was assessed in the SYNAPSE study, a multicentric randomized, double-blind, placebo-controlled, parallel-group, and phase 3 trial (31). According to the results of this study, mepolizumab represents an effective add-on treatment option to standard of care for CRSwNP. In particular, 414 patients enrolled in this study were randomly assigned (1:1) to receive mepolizumab subcutaneously (100 mg) or placebo once every 4 weeks in addition to standard of care (mometasone furoate intranasal spray, saline nasal irrigations, systemic corticosteroids or antibiotics, or both), as required. At week 52 from baseline, endoscopic nasal polyp score and nasal obstruction VAS score were significantly improved in the mepolizumab group compared to the placebo one.

**Reslizumab** is another humanized monoclonal antibody approved in Europe and the USA for adult patients as add-on maintenance treatment for severe asthma with an eosinophilic phenotype (32). Reslizumab binds IL-5 with a picomolar affinity, reducing consequently survival and activity of eosinophils (33). Regarding its efficacy in nasal or sinus mucosal diseases, few data are available. In 2016, a first double blind, randomized, placebo-controlled, phase III trial started in California with the purpose of determinating whether reslizumab treatment was effective also for the chronic sinusitis. To date, although the study has passed its completion date, its status on Clinicaltrial.gov database results unknown (34). Another study evaluating the efficacy for the chronic rhinosinusitis symptoms in asthma patients undergoing reslizumab treatment was conducted in United States by the Department of Otolaryngology Head and Neck Surgery, University of Rochester. Its primary objective was to monitor the CRS symptoms in this patient population. This was a prospective observational study started in 2017, but subsequently withdrawn (34). Finally, a third mAb targeting on anti-IL5 pathways, **benralizumab**, is actually authorized in Europe as an add-on treatment in adults with eosinophilic asthma inadequately controlled despite high-dose inhaled corticosteroids plus long-acting β-agonists. This humanized monoclonal antibody targets IL-5Rα with high affinity and specificity. The IL-5 receptor is specifically expressed on the surface of eosinophils and basophils. Benralizumab reduces eosinophilic inflammation by inducing the apoptotic process of eosinophils and basophils, through enhanced antibody-dependent cell-mediated cytotoxicity (ADCC)^3^. Recently, a sub-analysis of the Phase IIIb ANDHI trial has been published, whose results extend benralizumab's efficacy to severe eosinophilic asthma patients with comorbid NP (any severity) (35). In particular, improvements in the annualized Sino-Nasal Outcome Test-22 (SNOT-22), asthma exacerbation rate (AER), FEV 1, Asthma Control Questionnaire 6 (ACQ-6), and St. George's Respiratory Questionnaire (SGRQ) total score were observed with benralizumab treatment compared to placebo. Likewise, benralizumab efficacy and safety profile in patients with severe NP was confirmed by the results of another randomized, double-blind, placebo-controlled trial conducted by Tversky et al. For this study, 24 patients with severe NP (defined by endoscopic grade 5 or more out of 8) and elevated eosinophils, with a history of previous surgical or endoscopic polypectomy, were enrolled. Benralizumab achieved a statistically significant reduction in nasal polyp size, sinus occupancy, symptoms and improved sensation of smell for 83% of patients. Moreover, it was well-tolerated (36). Recently, Humanitas Clinical and Research of Rozzano Hospital (Milan, Italy) conducted a pilot, prospective, double-blind, placebo-controlled, phase III-b trial in order to assess benralizumab clinical efficacy after week 24 of treatment. For this study, benralizumab 30 mg was subcutaneously administered in patients with CRSwNP (allergic and non-allergic), every 4 weeks for the first 3 doses and then every 8 weeks. Moreover, in order to identify any possible predictive biomarker of response, an inflammatory e molecular phenotyping of responders to benralizumab was performed. Its results have been included in the aforementioned Cochrane Reviews, Biologics for chronic rhinosinusitis (16).

#### mAbs Targeting the IL-4/IL-13 Pathway

IL-4 and IL-13 are two Th2-associated cytokines with a mutual and important role in the type 2 inflammation. They share a same heterodimeric receptor, consisting in the combination of two subunits, IL-13Rα1 and IL-4Rα chain. This can be activated by both IL-4 and IL-13 (13). In particular, IL-4 and IL-13 pathways induce effects on keratinocytes (impairing their differentiation), eosinophils (inducing their activation), fibroblasts (increasing the production of eotaxin), B cells (IgE production), Th2 cells (increased the differentiation and survival). They play a fundamental role in the pathogenesis of nasal polyposis. Dupilumab, binding to IL-4Rα, blocks signaling of both the IL-4 and IL-13 pathways, resulting in a powerful inhibition of Th2, eosinophil recruitment, and IgE production. **Dupilumab** is a completely human mAb, administered as a subcutaneous injection every 2 weeks. Initially dupilumab has been authorized for the treatment of asthma and atopic dermatitis, as Th2 mediated diseases. Subsequently, it was also approved, first by the FDA (in June 2019) and then by the EMA (November 2019), as the first biological medicine for the treatment of inadequately controlled CRSwNP in adult patients. These authorizations were based on the results of two Phase 3 studies, SINUS-24 and SINUS-52 studies, which evaluated the effects of dupilumab administration (300 mg) every 2 weeks plus intranasal corticosteroids compared to placebo plus intranasal corticosteroids, at 24- and 52-weeks, respectively (37). According to the results of these studies, dupilumab significantly improved the signs and symptoms of severe CRSwNP. In particular, it induced improvements in nasal polyp size, sinus opacification and health-related quality of life (HR-QOL). The major symptoms of CRSwNP, including nasal congestion or obstruction, nasal discharge and loss of smell, were relieved. Moreover, it allowed a reduction in the use of systemic corticosteroids and nasal polyp surgery, being generally well-tolerated. Furthermore, dupilumab has also been shown to improve lung function in asthmatic patients. It is important to highlight this result since many patients with CRSwNP also suffer from asthma. Recently, Laidlaw et al. reported the results of a randomized, double-blind, placebo-controlled trial according to which dupilumab improved both upper and lower airway outcome measures and HRQoL in patients with severe CRSwNP and comorbid asthma. This study also confirmed its positive tolerability profile. The most common adverse events were nasopharyngitis, headache, injection-site erythema, worsening of nasal polyposis, and asthma. These were more frequent with placebo group than dupilumab (38).

Given the high prevalence of chronic respiratory diseases and the high cost asscociated with biological products, patient selection is crucial. During the European Forum for Research and Education in Allergy and Airway Diseases (EUFOREA) in 2019, a multidisciplinary Expert Board proposed indications for biological treatment use in CRSwNP. Some indicative criteria for biological treatment in CRSwNP patients were identified. These included evidence of type 2 inflammation with biological biomarkers, need for systemic corticosteroids (2 or more courses in the past year), significantly impaired quality of life, significant loss of smell, or diagnosis of comorbid asthma. Based on a previous history of surgery, the use of a biological treatment was suggested in patients with presence of bilateral nasal polyps if 3 or 4 aforementioned criteria are found, respectively (39). Moreover, **lebrikizumab** and **tralokinumab** are other two antibody therapeutics that prevent binding of IL-13 to its receptors. In particular, lebrikizumab targets IL-13 with high-affinity, preventing the formation of the IL-13Rα1/IL-4Rα heterodimer receptor signaling complex. Since lebrikizumab does not prevent the binding of IL-13 to the IL-13Rα2 receptor, it does not interfere with the endogenous regulation of IL-13 (40). Actually, they have been investigated only in other pathologic conditions, in particular asthma (including allergic type) and atopic dermatitis. No trials in sinus or nasal cavities diseases have yet been conducted.

### Innovative Drugs for the Treatment of Oncologic Rhinological Diseases

The nasal sinus neoplasms consist of a heterogeneous group of benign or malignant tumor histotypes which require different diagnostic-therapeutic management. Among the benign forms, the sinonasal hemangioma represents a rare vascular-type tumor of endothelial cells. Recently, a case report describing the administration of bevacizumab (50 mg) as treatment for a recurrent sinonasal hemangioma has been reported in the literature. The administration was performed by intralesion injection under endoscopic visualization in a 67-year-old patient. After 10 months, a reduction in the tumor size, a complete resolution of epistaxis and nasal obstruction were observed (41). Bevacizumab is a mAb that, by binding the growth factor of vascular endothelial cells (VEGF), blocks its biological activity. It is indicated as treatment for several types of solid tumor. It induces regression of the tumor vascularization, inhibits the formation of new vascularization, with consequent arrest of tumor growth (42).

The major rhinologic field of application of innovative drugs is **nasopharyngeal carcinoma** (NPC). This is a rare type of head-neck cancer. There are ~129,000 new cases of NPC each year worldwide. Over 70% of such cases are reported in South China and Southeast Asia. This tumor is etiologically associated with the Epstein-Barr virus (EBV). It represents an “inflamed tumor” archetype, showing often a dense lymphocytic infiltrate and increased expression of the programmed death ligand (PD-L1) (43). For this reason, the patients with NPC are potentially suitable for treatment with immune checkpoint inhibitors (ICIs). The ICIs are newly introduced mAbs that have literally revolutionized the treatment of several solid tumors. Cancer cells are able to evade recognition and subsequent elimination by the immune system through a series of adaptive responses, including the overexpression of various immunosuppressive molecules in the tumor microenvironment. Some of these molecules, such as CTLA-4, PD-1, and its PD-L1, are targets of ICIs. By blocking these immunosuppressive molecules, ICIs induce the reactivation of cytotoxic T lymphocytes able to destroy cancer cells. ICIs treatments showed significant clinical benefit for different types of cancer, establishing immunotherapy as an important advance in cancer treatment (44). In order to evaluate the efficacy in nasopharyngeal carcinoma of some anti-PD1 agents, such as pembrolizumab, nivolumab, camrelizumab, several clinical studies were conducted. Other ones are still in progress (43). Among ICIs, **nivolumab** has promising activity in nasopharyngeal carcinoma. Recently, the results of an international phase 2 study evaluating the antitumor activity of nivolumab in the treatment of NPC were published. This study was conducted in 44 patients with pre-treated recurrent or multiple metastatic NPC treated with nivolumab until disease progression. A complete response was observed in one patient, while eight patients showed a ≥30% decline in tumor dimension, defined as partial response. The disease control rate was 54.5%. The 1-year overall survival rate was 59% (95% CI, 44.3–78.5%) and the 1-year progression-free survival (PFS) rate was 19.3% (95% CI, 10.1–37.2%) (45). Recently, the findings emerging from a first phase 2 study of ipilimumab/nivolumab combination in NPC were presented in the context of ESMO Asia Virtual Congress 2020. According to these results, this ICI combination provide durable responses in patients with recurrent or metastatic NPC (46).

## Technological Innovation

### Drug-Eluting Implants

Various types of devices are available for nasal drug delivery systems. Biomaterials and sinus implant are some of these. Thanks to the incessant progress of technology, new biomaterials and sinus implants have been investigated, providing postoperative effective local corticosteroids into the sinuses. Over the years, the biomaterials have been used in the CRS post-operative management settings. Polylactide sinus implants, polyurethane foam, and carboxymethylcellulose were commonly used biomaterials (47). The **bio-absorbable implants** represent an example of innovative pharmaceutical technologies. In particular, these implants allowing local release of corticosteroids (CS) could be useful in the post-operative management of endoscopic sinus surgery (ESS). In patients with CRS, even more with CRSwNP, postoperative wound healing following ESS is an important factor for procedural success. After surgical treatment, topical or systemic CS therapy, and revision surgery are the available treatment options. However, these latter have significant risks and limitations. The topical nasal CS therapy ensure more effective and lasting symptomatic benefits, as well as reducing the size and number of polyps and preventing polyp recurrence. However, the distribution of topical steroids in the nasal cavity and sinuses is highly variable, depending on the delivery device as well as on the anatomy of the sinus drainage pathways. Steroid-releasing bio-absorbable implants have been extensively investigated for their ability to dilate and restore patency of the sinus by local and controlled release of CS. In the literature different CS-releasing bioabsorbable implants are described. A bio-absorbable, fluticasone propionate (FP)-eluting implant (SinuBand FP) resulted well-tolerated and effective in patients with CRS and nasal polyps. In particular, the results of a first-in-human, randomized, partially double-blind, single-tertiary-referral-center, controlled trial showed its local, and ocular safety. Compared to a standard nasal pack, or to a SinuBand without FP, SinuBand FP allowed significantly better polyp score (*p* = 0.03) and a better trend of inflammatory process. Patients receiving the bioabsorbable, fluticasone propionate-eluting implant reported lower pain (48). Moreover, bioabsorbable mometasone-eluting implants were also investigated (49, 50). In a prospective, randomized, double-blinded, placebo-controlled study, the endoscopic appearance in the healing process of CRSwNP after ESS was improved in patients receiving mometasone furoate (MF)-impregnated biodegradable nasal dressings (BNDs) (51). A comprehensive, up-to-date literature review reported a novel, mometasone furoate (MF) sinus implant such as useful treatment for patients with recurrent CRSwNP after ESS, playing an important role in the management (52). MF implants were also evaluated with additional topical nasal spray therapy. According to the results of a pooled analysis of data from 2 randomized controlled trials (RCTs), it emerged that this association has allowed more favorable results, in terms of subject than objective endpoints, compared to topical therapy with nasal spray alone, being useful in the management of patients with NP, especially those who have allergic rhinitis, expanded polyposis, altered odor or ESS <24 months (53). So, according clinical evidences, steroid-eluting bioabsorbable implants result safe and effective in the reduction of polyp size, symptom burden, and the need for revision sinus surgery. Favorable safety profile and efficacy of bioabsorbable steroid-impregnated implants in improving the healing process following ESS emerged from a recent meta-analysis including eight randomized controlled trials (54). About 14% of CRS patients undergoing surgery require ESS revision for a variety of reasons, including recurrence of nasal polyps and inflammation, adhesion formation, middle turbinate lateralization. So, the use of the nasal bioabsorbable implants appears to have a favorable economic impact. In fact, considering the substantial annual revision ESS costs, the use of the implant instead of revision ESS could result in considerable cost savings (55). **Sinus implants** made up of bioabsorbable polymers represent another new method to optimize surgical outcomes and to treat recurrent nasal polyposis after ESS. They allow sustained-release corticosteroids to be delivered locally directly to inflamed sinus tissues. Once implanted, these expand to fit different sizes and shapes, adapting to the space after surgery (47).

### Super-Selective Intra-Arterial Infusion of Chemotherapy With Concomitant Radiotherapy

Another innovative technological approach emerged also for the treatment of the maxillary sinus cancer (MSC). It is represented by the super-selective intra-arterial infusion of chemotherapy with concomitant radiotherapy (RADPLAT). It was developed in order to overcome some patients' problems related to advanced MSC surgical procedures, such as impaired facial function and significant facial deformity. Moreover, it is useful therapeutic strategy also for those patients with stage T4b MSC for which there is no indication of surgical resection (56, 57). The super-selective intra-arterial chemotherapy with radiation therapy reflects a precision medicine approach associated with a low risk of side effects. This effective procedure might be useful to avoid highly invasive surgery (58). However, the published studies refer to small patient samples (56–59). The introduction of technological innovations has allowed a significant expansion of outpatient rhinology (60).

### Balloon Catheter Dilatation

Innovation refers also to improved old technologies, like balloon-dilatation. Since 2005 the balloon catheter dilatation (BCD) represents a useful intervention for the management of CRS. BCD is a minimally invasive procedure aimed to restore physiological sinus drainage, safely dilating sinuses through microfractures (61). BCD has become a common treatment for chronic sinusitis in the United States (62). It is among the most common office-based rhinological procedures (60). Over the years, it has been renewed in several aspects, including innovations in ergonomics and lighted guidewires in order to make the utilization more effective and safer. Now, the new devices are equipped with suction and irrigation capabilities and allow multisinus applications using just one device. Moreover, the previous tools used fluoroscopy for localization with the consequent risks of exposure to radiation. Today, transillumination and real-time 3-dimensional image guidance have been introduced to overcome these problems. Some studies suggest the use of BCD as a safe tool in the management of pediatric CRS (pCRS). However, they refer to small samples and show methodological limitations (63–65). According to recent meta-analyses and systematic reviews, more evaluations are needed to demonstrate its clinical usefulness in terms of improving the quality of life and the comparative efficacy of BCD compared to standard treatment regimens in specific patients' subgroups such as children (61, 65–67). Moreover, as with all interventions, BCD, although minimally invasive, can be associated with adverse events such as cerebrospinal fluid leaks, mainly reported with frontal sinus procedures (62).

### Artificial Intelligence and Machine Learning

In the era of big data, the application of artificial intelligence (AI), the machine learning (ML), and particularly deep learning, represent increasingly relevant topics of the health care research, also in the rhinology field. Recently, in order to efficiently use all recorded data, AI and ML technology has been used in some studies of chronic rhinitis and allergic rhinitis, providing some exciting new research modalities. Regarding the application of AI and ML technology, few reports describe their use in rhinology. Only recently (since 2015) a slow increase in their descriptions emerged in literature. In the majority of the rhinologic studies in which an AI approach was used, cluster analyses were performed, i.e., to predict surgical vs. medical treatments for CRS in patients who did not have successful outcomes after initial medical treatment (68). Regarding the ML technology, the majority of algorithms are divided into supervised or unsupervised learning. This latter has been reported as a novel tool in the investigation of CRS. It represents a paradigm shift from the traditional approach based upon the clinically recognized phenotypes of CRS “with polyps” and “without polyps.” Instead, an unsupervised learning approach using the application of complex mathematical models is able to derive other different subgroups which can then be further examined (69).

### Augmented and Virtual Reality

Finally, also the augmented reality (AuR) and the virtual reality (VR) represent other new technological approaches applied to the rhinology field. The main difference between these new technologies consists in an enhancement of a user's natural vision obtained with AuR, that instead, in VR, comes completely replaced. Today, the VR can be used in the surgical simulation, allowing modernization of training and its transition from the practice of simple exercises into a fully-immersive environment experience. In a recent study, the use of a virtual coach was tried guiding a group of surgeons using surgical videos, auditory, and visual cues (70). In AuR, the real-world environments are combined with computer-generated sounds, text, and graphics. So, the AuR represents a tool for the surgeons that improve visualization, location, and orientation allowing improvement of surgical outcomes in terms of operating time, precision, and increased surgeon confidence. AuR can also represent a tool for procedure simulations or anatomy education, allowing the students to learn head and neck anatomy, often difficult to conceptualize. It has grown rapidly and continues to expand (71). Rarely, the AR has been used as a diagnostic and treatment tool through specific AuR-based platforms described in some studies.

## Digital Innovation

Today, we are in the digital era. Digital tools and devices are ubiquitous. We are learning to exploit the goals achieved in terms of connectivity and connection also for the management of health and, therefore, of diseases. Thanks to the achieved technology progress, therapeutic treatments can take advantage of software and devices. The digital approach is particularly able to obtain a real-time control and support of behavior and health status, improving quality of health care in the long term by greater patient involvement. We are witnessing to the introduction of **digital therapeutics**, as clinically validated treatments designed to complement or potentially replace traditional therapies (72). Moreover, digital advances have allowed innovative, almost futuristic approaches such as that of **digital twins**. These latter represent an engineering concept which can be applied to different complex systems, including that of human physiology (73). Digital twins are built on computer-based models that are fed individual and population data. The translation of the digital twin concept to patients aims to improve diagnostics and treatment in order to deliver data-driven personalized medicine. Beyond these revolutionary paths, digital progress is applied even to innovative approaches that are much more accessible. These take the form in eHealth, mobile-Health (mHealth) such as the telemedicine based on the obtained connectivity of mobile devices with the internet (74–77). Today, given the current COVID-19 pandemic, several professional societies are encouraging the maximization of the use of telemedicine in current practice (78). This approach introduced a new way for generating health and medical data—by the individual, in real time, in a real-world environment. Although these features are interesting, the benefits of digital medicine have to be proven through rigorous research, especially validation through randomized, controlled clinical trials. The **mHealth**, as a branch of eHealth defined as “medical and public health practice supported by mobile devices,” can be effective to facilitate communication between primary care providers, able to overcome geographical and temporal barriers as well as to treatment accessibility and availability. It can use different tools, including smartphone applications (app), SMS text messaging with a support service, physical symptom tracking through wearable technologies, and receiving virtual therapy. The mHealth tools are developed to improve patient empowerment *via* education and self-management and will hopefully contribute to better patient adherence, quality feedback to the physician and improved patient health literacy. As reported by mHealth users, it is advantageous compared with face-to-face therapy, allowing them to be more open and honest (79). Moreover, mHealth therapy allows rapid adaptation of the treatment strategy based on the symptoms, concomitant medications and key events that may impact the disease. In particular, mobile applications are achieving a prominent position in the management of chronic diseases. For chronic respiratory diseases, most of the apps have been developed for lower respiratory diseases such as asthma or COPD. Recently, Bodini et al. have identified 5 Digital Therapeutics (DTx) for asthma and COPD which combine sensor devices, mApps for patients, and cloud-based software for healthcare professionals (80). They consent to record if/when/how the patient uses the inhaler, to alert for use the inhaler, to receive information from the sensor, providing a personalized support and remote monitoring. To date, mySinusitisCoach is a mobile app available for patients with sinus disease (81). This tool has been launched during the European Rhinology Research Forum (ERRF) 2017. It was designed, developed and implemented to support CRS patients in monitoring their symptoms and to provide patients with a digital support platform containing reliable medical information about their disease and treatment options. MySinusitisCoach has been developed thanks to a collaboration between CRS medical experts, patients, general practitioners and community pharmacists. This collaboration was sought to obtain a tool that would meet the needs of both patients and healthcare professionals. Its functionalities include the monitoring of symptoms and consumption of drugs, the visualization of the disease control level, providing unbiased information on chronic sinusitis and asthma. Moreover, the easy sharing of data with the doctor in order to obtain a real-time connection between the patients and health workers, allows optimization of treatment. Recently, a cross-sectional evaluation of data obtained by users of mySinusitisCoach. This real-life assessment confirms the high disease burden in uncontrolled CRS patients, which can be supported by mobile technology in the real-life monitoring (82). The mobile apps allows not only a continuous and remote monitoring of the patient's health status, but also an important collection of real-world data that will help in clinical studies validating patient stratification as well as understanding of the socio-economic impact of CRS, in order to improve treatment strategies. Recently, mySinusitisCoach has been replaced by Galenus Health, a mobile app developed by a team of internationally recognized doctors designed for anyone with asthma, respiratory allergy or chronic sinusitis, often concomitant diseases. Finally, the use of digital approach with a smart language can also be used for the improvement of patient education. In fact, its use can positively impact on patient outcomes such as anxiety, pain and satisfaction in relation to the perioperative patient experience. Online education materials are often too complex, inaccurate or misleading to be useful to the patient. A recent study has been conducted by University of British Columbia in order to evaluate the effect of patient education videos on perioperative anxiety in patients undergoing endoscopic sinus surgery. The enrolled patients received four short YouTube videos explaining chronic rhinosinusitis and endoscopic sinus surgery. Patients of the control group received the standard of care patient education with verbal and written education. The study is completed but results not yet available (83, 84).

## Conclusion

**Figure 3:**
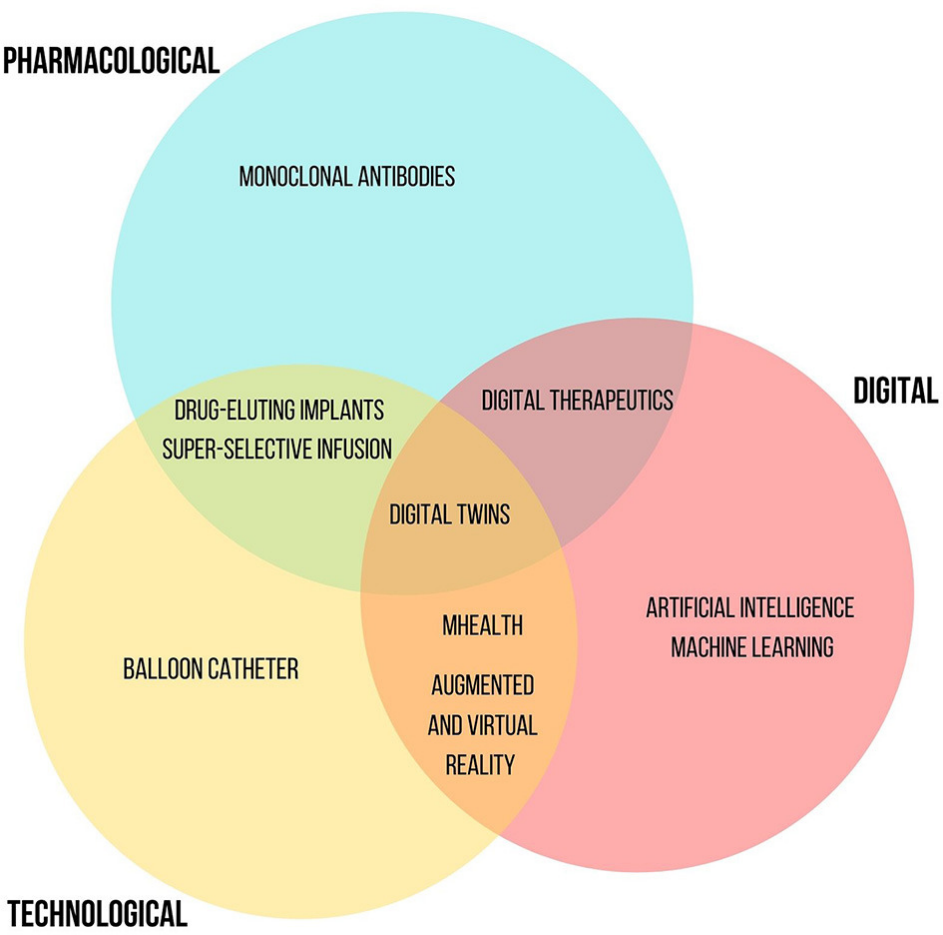
Overlapping nature of each innovative tool in Rhinological conditions.

Innovative aspects in rhinology involve new drugs, technologies for their administration as well as digital applications. Each different innovative tool has an important impact and allows an improvement in several clinical and patients outcomes, including quality of life, efficacy and safety. The discussed innovative tools show an overlapping nature among the considered fields (Figure 3). The emerging innovative drugs include mAbs targeting on characteristic pathways of type 2 inflammation, such as those of IgE, the IL-5, and IL-4/IL-13 which are involved in several pathologic condictions including CRSwNP or allergic rhinitis. Dupilumab (anti IL-4), mepolizumab (anti IL-5), and omalizumab (antiIgE) represent the main mAbs developed such as innovative treatment options for patients with NP and CRS. They seem to allow improvement in terms of quality of life, disease severity and tolerability of treatment. Other mAbs are in the advanced research stages like etokimab (anti-IL33) or tezepelumab (targeting on TSLP). Moreover, another important application field of mAbs is the oncological immunotherapy. Considering the higher expression of PD-L1 in NPC, the use of PD-1 inhibitors, such as nivolumab, or a dual CTLA-4/PD-1 blockade (85) appear to be an effective strategy for the treatment of this cancer form. However, current studies are not yet at in advanced stages. The careful monitoring of patients with regard to the autoimmune toxicity related to them should not be underestimated. Regarding the technological innovation, the implants with bio-absorbable biomaterials represent new interesting available technologies. In particular, those allowing the topical administration of corticosteroids drugs (fluticasone and mometasone) are useful treatments for patients with recurrent CRSwNP after ESS, playing an important role in its management. Moreover, considering the substantial annual costs of ESS, their favorable economic impact is worthy of note. Advanced technologies such as, AI and ML, as well as AuR and VR have also proved useful in the rhinologic field with their main impacts on precision medicine and surgery. Finally, the development and use of mHealth tools represent a winning strategy in monitoring therapy success, safety and tolerability as well as the progress of chronic disease including CRSwNP. They seem to be efficient and effective mainly in the improvement of patients' outcomes. The mobile apps allow to improve patient empowerment by an active participation in the decision-making process of the therapeutic plan. Likewise, their use allows collection of real-world data that will help to improve treatment strategies in a greater perspective of personalized and precision medicine. So, supporting the research of innovative tools and strategies (including pharmacological, technologic, or digital ones) is essential to improve the management of chronic diseases that significantly affect the quality of life of patients. Further studies are strongly needed in order to support their use in real life context. In the future, the use of combined innovative approaches is desirable.

## Author Contributions

FR, AC, and RR: drafting the work and revising it for important intellectual content. RR, GioM, and GiuM: substantial contributions to the acquisition, analysis, or interpretation of data for the work. FR, CR, and ADC: final approval of the version to be published. AC, AD, and GaM: agreement to be accountable for all aspects of the work in ensuring that questions related to the accuracy or integrity of any part of the work are appropriately investigated and resolved. FR and GaM: developed the concept and designed the study. RR and GioM: wrote the paper. All authors contributed to the article and approved the submitted version.

## Conflict of Interest

The authors declare that the research was conducted in the absence of any commercial or financial relationships that could be construed as a potential conflict of interest.

## Publisher's Note

All claims expressed in this article are solely those of the authors and do not necessarily represent those of their affiliated organizations, or those of the publisher, the editors and the reviewers. Any product that may be evaluated in this article, or claim that may be made by its manufacturer, is not guaranteed or endorsed by the publisher.

## Footnotes

^1^CHMP. *Annex I Summary of Product Characteristics*.

^2^Omalizumab (2021). Available online at: http://www.ncbi.nlm.nih.gov/pubmed/30000860 (accessed April 23, 2021).

^3^Fasenra and European Medicines Agency. (2021). Available online at: https://www.ema.europa.eu/en/medicines/human/EPAR/fasenra#product-information-section (accessed April 30, 2021).
